# Protective Effects of Salicylic Acid and Calcium Chloride on Sage Plants (*Salvia officinalis* L. and *Salvia* *elegans* Vahl) under High-Temperature Stress

**DOI:** 10.3390/plants10102110

**Published:** 2021-10-05

**Authors:** Kuan-Hung Lin, Tse-Yen Lin, Chun-Wei Wu, Yu-Sen Chang

**Affiliations:** 1 Department of Horticulture and Biotechnology, Chinese Culture University, Taipei 11114, Taiwan; rlin@ulive.pccu.edu.tw; 2 Department of Horticulture and Landscape Architecture, National Taiwan University, Taipei 11106, Taiwan; est8650602@yahoo.com.tw; 3 Department of Horticulture, Hungkuo Delin University of Technology, New Taipei 23630, Taiwan

**Keywords:** calcium chloride, chlorophyll fluorescence, high temperature, heat stress, salicylic acid, spectral reflectance, *Salvia officinalis* L., *Salvia elegans* Vahl

## Abstract

High-temperature stress is a major risk to fresh-market *Salvia* production, and heat intolerance is a major constraint in sage cultivation, particularly during the hot summer season. Previously, we investigated heat tolerance in five common-market cultivars of sage plants using leaf relative injury (RI) values and found that *S. elegans* Vahl (SE) and *S. officinalis* L. (SO) were the most and least heat-tolerant species, respectively. The exogenous applications of salicylic acid (SA) and calcium chloride (CaCl_2_) to alleviate heat stress in various species have been extensively studied, but reports of the effects of SA and CaCl_2_ treatments on the heat tolerance of sage plants are scarce. The objective of this study was to investigate how SA and CaCl_2_ affect the physiology and morphology of SE and SO plants under high-temperature conditions. Potted plants were pretreated with SA (0, 100, 200, 400, and 800 μM) and CaCl_2_ (0, 5, 10, and 15 mM), alone and combined, exposed to 55 °C and 80% humidity for 30 min, then placed in an environment-controlled chamber at 30 °C for three days and evaluated for changes in phenotypic appearance, RI, spectral reflectance, and chlorophyll fluorescence indices at different time intervals. Plants watered without chemical solutions were used as controls. Our results show that the growth of SO plants pretreated with SA and CaCl_2_ was more robust, compared with control plants, which were considerably affected by heat stress, resulting in brown, withered leaves and defoliation. The effects of the combined applications of SA (100 μM) and CaCl_2_ (5 mM) to SO plants were superior to control plants in increasing values of soil-plant analysis development (SPAD), normalized difference vegetation index (NDVI), and the maximal quantum yield of photosystemII photochemistry (Fv/Fm), while reducing RI%. Furthermore, SO plants exhibited higher SPAD and Fv/Fm values and lower RI% than SE plants in combined treatments at all time intervals after heat stress, implying that different genotypes displayed variations in their SPAD, Fv/Fm, and RI%. Thus, a combined treatment of 100 μM of SA and 5 mM of CaCl_2_ is effective and beneficial to plant appearance and ability to ameliorate heat stress. These indices can be used as indicators to characterize the physiology of these plants and applied on a commercial scale for informing the development of rapid and precise management practices on bedded sage plants grown in plant factories to achieve maximum market benefit.

## 1. Introduction

*Salvia* is a member of the Lamiaceae, a family represented by 45 genera. Its fragrant plants are used in folk medicines around the world, and many of its species are important in the pharmacology, perfume, cosmetics, and food industries because they contain volatile and aromatic oils [1,2]. *Salvia* is native to Middle Eastern and Mediterranean regions and contains more than 900 species spread throughout the tropical and temperate regions of Europe and Asia [3]. Several reports indicate that leaf essential oil extracts of *S. officinalis* L. (SO) have specific spasmolytic, astringent, antimicrobial, antihidrotic, antioxidant, and hepatoprotective effects, in addition to sensory, antigenotoxic, and chemopreventive activity [4,5,6]. These plants areherbaceous perennial ornamentals. They are encountered in many areas and regions in Taiwan where daily temperatures exceed 33 °C in summer from June to September, resulting in heat stress, inhibited plant growth, and poor quality and quantity. Currently, no method, apart from field observations, has been developed for phenotyping high-temperature susceptibility in garden sage plants; thus, the ability to display fresh-market sage production is of great importance commercially. There is considerable pressure on the floriculture industry to produce ornamental plants more efficiently, and as the landscaping demand for sage plants is increasing, any method to improve heat tolerance in the hot summer season is important to Taiwan nurserymen. Moreover, understanding sustainable heat tolerance informs assessments of performance and the adaptation of sage plants to certain growing areas, breeding programs, and scheduling of culture techniques and management. The responses of *Salvia* species to water stresses [7,8], salt stresses [9,10], and ozone-induced oxidative stresses [11] are reported, but there is limited information available regarding the morphology and physiology of sage plants grown under high-temperature stress.

Plants have been developed with different adaptive mechanisms to cope with heat stress by inducing physiological responses; hence, understanding the physiological mechanisms of resistant *Salvia* species in response to heat stress is important. Heat stress reduces the ability of photosynthesis to utilize incident photons leading to photoinhibition, a concomitant reduction in the quantum yield of photochemistry, and a decrease in chlorophyll fluorescence (ChlF). ChlF measurements, such as the maximal quantum yield of PSII photochemistry (Fv/Fm), is a noninvasive technique that has been widely used in a range of photosynthetic organisms and tissues to study functional changes in the photosynthetic apparatus under different heat stress conditions in controlled environments and in the field [12]. Reflectance spectra are altered when stress occurs, and these alterations can be used to calculate different vegetation indices such as the adjusted normalized difference vegetation index (NDVI), which has been linked to photosynthetic light-use efficiency [13]. The soil-plant analysis development (SPAD) meter assesses total Chl contents and photosynthetic capacity and is widely used for the rapid, accurate, and nondestructive measurement of Chl concentrations in leaves. The effective management of these parameters in response to chemical treatments provides a better understanding of the photosynthetic characteristics of sage plants grown in high temperatures.

Heat’s unfavorable effects can be alleviated by thermo-tolerance induced by the exogenous application of plant growth regulators and osmoprotectants, or by the gradual application of temperature stress. Both salicylic acid (SA) and calcium chloride (CaCl_2_) are recognized as signaling molecules for their roles in plant adaptation to changing environments, influencing various stress responses and regulating the physiological and biochemical mechanisms of plants adapted to adverse environmental conditions [14,15]. Applying SA to *S. nemorosa* under drought stress alleviates oxidative stress by maintaining cell membrane stability and enhancing photosynthetic capacity and antioxidant defense mechanisms [16]. In addition, several studies show the remedial effect of SA on the harmful effects of salinity on sage [17], strawberry [18], and corn [19], and attribute the positive effects of SA under saline stress in improving growth by stimulating photosynthetic capacity and protecting performance parameters. Palta [20] found a decrease in Ca^2+^ concentration in potato leaves exposed to heat stress, but the Ca^2+^ concentration during heat stress in the leaves of plants pre-applied with CaCl_2_ was maintained at the same level as before the heat stress, suggesting that holding a certain Ca^2+^ level in guard cells to maintain normal stomatal function allows plants to avoid heat stress effects by dissipating heat through transpiration. The combined applications of SA and CaCl_2_ improve growth and mineral nutrition in tomatoes during salt stress [21]. Studies of SA and CaCl_2_, individually or combined, in wheat under salt stress reveal that combined applications proved more effective in reducing oxidative stress [22]. Furthermore, the use of SA and CaCl_2_ protected *Rhododendron* from injuries induced by heat stress [23]. Lateral bud sprouting and new leaves in poinsettia (*Euphorbia pulcherrima* Willd.) cultivars were increased by SA and CaCl_2_ applications in response to heat stress as a result of enhanced catalase activity and reduced malondialdehyde (MDA) levels [24]. However, only a limited number of studies have investigated the heat resistance of sage plants, and the influence of SA and CaCl_2_ pretreatment on sage heat tolerance has never been reported.

There are several physiological stress markers that can be measured in plants to provide insight as to how cell membranes behave under heat-stress conditions, and the percent relative injury (RI%) is one of the most commonly used parameters. The ability to quantify high-temperature stress and rapidly screen sage cultivars for high-temperature susceptibility is dependent on the temperature and duration of treatment. Previously, we analyzed and maximized the differences in RI% among five commercial sage cultivars for the differences in high-temperature tolerances among cultivars and optimizing the stress duration. We compared potted plants subjected to stress from 10 to 60 min intervals at 30, 35, 40, 45, 50, and 55 °C to maximize the differences in RI% among cultivars and achieve maximum separations based on cultivars having significantly different performances. In general, all differences detected between cultivars corresponded to appearance, with *S. elegans* Vahl (SE) having a superior performance over SO plants under 55 °C for 30 min, which was sufficient to detect differences among cultivars with known differences in summer landscape performances. None of the SE plants showed any visible signs of stress immediately after heat chamber treatment.

The hypothesis of this experiment was that the effects of heat stress on SE and SO genotypes could be lessened by pretreatment with SA and CaCl_2_ under growth chamber conditions because it might protect cell membranes from the adverse effects of heat-shock stress. Thus, we tried to determine how SA and CaCl_2_ affected the physiology and morphology of SE and SO species under an induced heat-shock stress, as their tolerance responses were directly linked to the coordinated responses of physiological parameters and resulted from the effectiveness of CaCl_2_ and SA in alleviating the inhibitory effects of heat-shock stress.

## 2. Results

### 2.1. Effects of SA Treatments on Physiological Performance and Appearance between Cultivars under Heat Stress

Table 1 shows the differential responses of both species toward ChlF and spectral reflectance values for various concentrations of SA treatment after 0, 1, 2, and 3d of heat stress, and showed significant differences between species. The SPAD values of heat-tolerant cultivar SE plants with all SA treatments at 2 and 3d intervals were significantly higher (range 33.08~37.12), compared with untreated controls (28.23 and 29.17), indicating that the SPAD values of SE were affected by SA treatments. Nevertheless, the SPAD values of heat-intolerant cultivar SO plants at 1 and 3d after100~400 μM of SA treatment (range 34.36~39.76) were significantly higher than in the 800 μM SA treatment and control plants (range 28.03~32.19), revealing that 100~400 μM of SA treatments increased SPAD values in SO plants at 1 and 3d after heat stress. When SA treatments were compared across days after heat induction, NDVI values in SE plants had significantly higher values in all SA treatments (range 0.67~0.76) after 0, 2, and 3d, compared with control plants (range 0.56~0.61). However, no significant differences in NDVI levels were observed in SO plants treated with SA at all heat-stress time durations, except for the 3d period after heat stress, in which SO plants treated by 100~400 μM of SA exhibited significantly higher NDVI value (range 0.61~0.63) than 800 μM SA treated and control plants (0.49 and 0.50). The Fv/Fm values of SE plants showed no significant differences among all treatments at all times after heat stress, except that a significantly higher Fv/Fm (0.18) in 100 μM SA-treated and control plants was observed, compared with other treatments (0.02~0.03) at the 3d period. However, SO plants with 100 and 200 μM SA treatments and control plants had significantly higher Fv/Fm levels (range, 0.60~0.71), compared with the 800 μM SA treatment (0.38) at the 3d period.

Table 2 shows that RI% increased as SA concentration increased, and RI% of SE plants in 100 μM and 200 μM SA treatments were significantly lower (30.6% and 36.5%, respectively) than in the 800 μM SA treatment and control plants (42.8% and 40.2%, respectively) after heat-shock stress. In addition, the RI values of SO plants in 100~400 μM SA treatments were significantly lower (range 26.8~36.5%) than 800 μM SA-treated and control plants (53.9% and 48.7%, respectively) after heat-shock stress, implying that 100~400 μM SA treatments can protect the plants from thermal injury. The RI% value in the SE cultivar control was lower (40.2%) than in the SO cultivar (48.7%), indicating that SE was more heat tolerant than SO. RI% makes it easy to detect differences between cultivars in response to heat stress.

**Figure 1:**
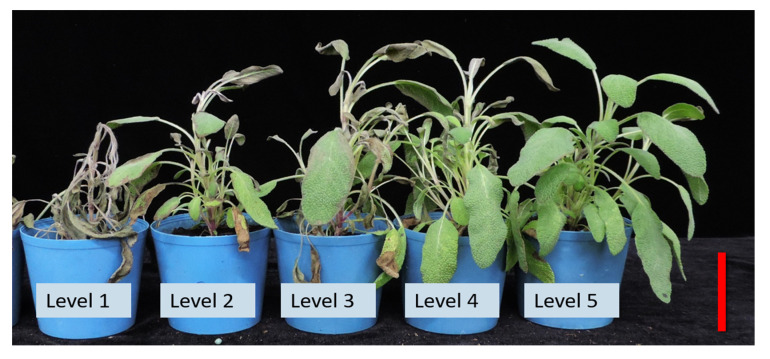
Morphological appearance of tested plants rated as 1~5 points based on the level of heat-induced injury. Level 1 (1 point): all leaves had become brown, withered, and abscised. Level 2 (2 point): >60% of leaves had become brown, withered, and abscised. Level 3 (3 point): 30~60% of leaves had become brown, withered, and abscised. Level 4 (4 point): <30% of leaves had become brown and withered. Level 5 (5 point): normal growth. The red bar indicates 5 cm.

Morphological quality was visually rated for plants in each pot on a scale of 1 to 5, with 5 indicating the best in all quality components and 1 indicating mostly brown, withered, and defoliated leaves (Table 3 and Figure 1). None of the SE control plants or those exposed to SA applications showed any visible signs of stress after heat treatment, ranging from 4.2 to 5.0, except that the 800 μM SA-treated plants showed significantly lower appearance grades (3.9), compared with 100 μM and 200 μM SA treatments and control. Furthermore, the 400 μM and 800 μM SA-treated plants and the SO control plants exhibited significantly lower appearance grades (range 3.7~3.9) than the 100 μM and 200 μM SA treatments (4.3 and 4.8). Tested plants showed changes in plant appearance after being treated with SA and CaCl_2_ alone or combined 3d after heat stress (Figure 1).

### 2.2. Effects of CaCl_2_ Treatments on Physiological Performance and Appearance of Cultivars under Heat Stress

As shown in Table 4, various CaCl_2_ concentrations acted differently at different time intervals after heat stress, and SPAD values of SE plants with both 5 and 10 mM CaCl_2_ treatments were significantly higher (range 38.54 and 39.50) than controls (range 29.00~35.00) at 0, 2, and 3d. The trends in NDVI values of SE plants under CaCl_2_ treatments over time were similar to SPAD values; however, NDVI values for SE plants did not display significant differences in all CaCl_2_ treatments at all time periods (range 0.57~0.69). Nevertheless, the Fv/Fm values of SE plants tended to decrease with increases in the number of days after high temperatures, and Fv/Fm values of SE plants did not display significant differences in all treatments at all periods, except that plants treated with CaCl_2_ exhibited significantly higher Fv/Fm values (range 0.17~0.19), compared with untreated control plants (0.05) at the 1d period. Furthermore, significantly lower SPAD values of SO plants were found in CaCl_2_ treatments (range 33.33~36.19), compared with controls (38.96 and 39.19) at 1 and 2d periods of high temperatures. No significant differences in NDVI values of SO plants were detected in all treatments at all time periods, except that significantly higher NDVI values were detected in the 5 and 15 mM CaCl_2_ treatments (0.58 and 0.61, respectively), compared with control (0.48) after 3d of heat stress. In addition, at both 2 and 3d periods following high-temperature treatments, Fv/Fm values of SO plants with 5 and 15 mM CaCl_2_ treatments (range 0.42~0.58) were significantly higher than in controls (range 0.09~0.27), suggesting that each CaCl_2_ concentration was not necessarily equally significant in protecting SO plants against heat stress.

Table 5 shows that the RI values of SE plants exposed to heat stress were significantly lower in 5 and 15 mM CaCl_2_ treatments (32.5% and 38.9%, respectively), compared with control (42.1%), whereas the RI levels of SO plants notably decreased as CaCl_2_ concentrations increased, and significantly lower RI values occurred with all CaCl_2_ treatments (range, 34.6~46.1%), compared with control (56.7%) after heat stress. Higher RI values indicate more extensive cell membrane injury.

Table 3 shows that SO control plants had a significantly lower appearance value (3.0) than those having CaCl_2_ treatments (range 3.4~3.8), and considerable visual damage increases occurred with increasing concentrations, whereas none of the SE plants showed any visible signs of heat stress after heat treatment with CaCl_2_ applications, ranging 4.8~5.0.

### 2.3. Effects of Combined Optimized Treatments on Physiological Performance and Appearance between Cultivars under Heat Stress

In general, Table 1, Table 2, Table 4 and Table 5 display that SO plants treated with single chemical treatments of 100 μM SA and 5 mM CaCl_2_ performed better than other treatments as measured by SPAD, NVDI, Fv/Fm, RI%, and appearance (Table 3). The combined chemical treatment of 100 μM SA + 5 mM CaCl_2_ is considered an optimal economic treatment of SO plants for physiological measurements. Table 6 shows that the highest ChlF and spectral reflectance values in all tested plants were obtained 3 d following high-temperature treatment after simultaneous applications of SA and CaCl_2_.The combined exogenous application of SA and CaCl_2_ to SE plants significantly increased SPAD levels (35.48 and 38.45, respectively), compared with untreated control plants (24.93 and 28.56, respectively) at both 2d and 3d after heat stress, implying that 100 μM SA and 5 mM CaCl_2_ application could be linked with enhanced total Chl contents.

However, SPAD values of SO plants were significantly higher in combined SA + CaCl_2_ treatments (range 51.19~52.30), compared with control plants (range 31.58~36.70) from1~3d after heating. In addition, NDVI levels with simultaneous applications of SA and CaCl_2_ to SE plants also showed significantly higher (0.69 and 0.68, respectively) values, compared with control plants (0.53 and 0.42, respectively) 2d and 3d after heat stress. Table 6 also reveals that SO plants showed significant increases in NVDI values in combined SA and CaCl_2_ treatments (range 0.67~0.68), compared with control plants (range 0.41~0.53) from1~3d after heat stress. Nevertheless, no significant differences in Fv/Fm values were observed in either genotype in any treatment, except that both genotypes exhibited significantly higher Fv/Fm values in the combined chemical treatment, compared with control plants 3d after heat stress.

When different chemical treatments were compared, SE plants treated with 100 μM SA + 5 mM CaCl_2_ exhibited significantly lower RI (31.5%) levels than control plants (43.9%) after heat stress (Table 7). Furthermore, SO plants in response to SA + CaCl_2_ treatment also had significantly lower RI (25.7%) than control plants (39.2%) after heat stress.

The appearance scores for SO plants treated with a combination of SA and CaCl_2_ (4.3) were significantly higher, compared with plants treated with SA or CaCl_2_ alone (3.0 and 3.3) and controls (2.6), whereas none of the SE plants showed visible signs of heat stress after heat treatment with chemical applications, recording a range of 4.7~5.0 (Table 3).

## 3. Discussion

Sage plants are economically important ornamental flowers and are traded worldwide as potted plants. High-temperature stress is a major risk to fresh-market *Salvia* production in Taiwan, and heat intolerance is a major constraint in sage cultivation. Heat stress affects the phenotype of a plant, causing leaf etiolation and wilting; it also alters the anatomy, physiology, and photosynthetic capability of plants [25].Visual appearance is the most important criterion to be considered for ornamental plants, and the ability of sage plants to maintain visual quality can make this ornamental plant a potential candidate for re-vegetating public and private lands. Furthermore, in the floriculture industry, the need for heat-tolerant plant cultivars is increasing because of rising global temperatures. In trying to understand the responses to high temperature, leaf-monitoring photosynthetic parameters were identified and characterized after heat stress, and the effects of high temperature on the appearance and physiological characteristics of two sage cultivars were examined in this study. The experiment was conducted to test the efficacy of chemical applications for improving appearance by enhancing the tolerance capacity of plants against high-temperature stress, reduce the impact of heat stress during vegetative growth stages, and develop a strategy to improve the heat tolerance of sage plants. Comparing the appearance ratings of all tested plants, the SE genotype was more tolerant (range 3.9~5.0) relative to the SE genotype (range 2.6~4.8) in all chemical treatments and controls (Table 3), displaying normal growth and development (except in the 800μM SA treatment) under heat stress. SE plants tended to be unaffected and exhibited adaptive morphologic plasticity. In contrast, the leaves of untreated SO plants turned brown and withered, in particular, their lower leaves looked epinastic, chlorotic, and senescent, plants defoliated with different levels of injury and damage, and damage was irreversible. However, after chemical treatments, evaluations 3 days post heat stress also revealed no visible signs of high-temperature damage, e.g., shoot burn or leaf necrosis (Figure 1). Thus, SO plants treated with exogenous SA and CaCl_2_, alone or in combination were markedly protected from heat-induced growth inhibition. SO plant control groups had the lowest scores, compared with treatments, and a few of the SO control plants after 3d of high-temperature stress clearly showed heat stress phenotypes with differing levels of severity, including plant death, indicating the highest degree of injury among all treatments. Compared with single chemical treatment and controls, all combined chemical treatments induced more new leaves in the SO cultivar (photos not shown), suggesting that CaCl_2_ and SA acted as primary signaling molecules for regulating new leaves in response to heat stress. Further work needs to be conducted to confirm whether SA and CaCl_2_ have potential use for increasing the dry weight production of these sage plants.

As time passes following heat stress, the SPAD and NVDI values of all control plants displayed remarkable decreases, compared with plants treated with 100~400 μM SA (Table 1). In addition, the lowest and highest RI% values for all plants were detected in the 100 μM SA treatment and controls (Table 2). SO plant SPAD, NVDI, Fv/Fm, and RI values tended to be more sensitive to 100μMand 200 μM SA treatments than the 800 μM SA treatment. Although our study did not reveal a specific dosage for elevating SA in sage plants, it would appear that 100μMand 200 μM SA may be suitable. In addition, visual deterioration in SO plants increased as SA concentration increased (Table 3), and there were visually significant reductions in plant growth. Yadegari [26] showed that at low concentrations, while SA had a stimulating effect at higher concentrations, it reduced essential oil content in *S. officinalis*. Furthermore, Es-sbihi et al. [27] also reported that a low (0.5 mM) SA application to *S. officinalis* reduced plant Na^+^ content, improved growth, and increased nutrient (calcium, potassium, and phosphorus) levels, chlorophyll, essential oil content, and peltate gland density. SO plants are clearly highly tuned to the absolute levels of SA because a small amount of change can result in drastically different responses. Manipulating SA homeostasis by altering the concentration of SA could be an important strategy for altering the behavior and survival of SO plants under heat stress. As a consequence, SA may play an important role in the photosynthetic system under heat stress, but too high a concentration may destroy the photosynthetic ability to remediate leaf damage. In addition to SA application, significantly higher SPAD but lower RI values of SE plants were detected in the 5 mM CaCl_2_ treatment, compared with controls after heat stress, and significantly higher NDVI and Fv/Fm but lower RI values for SO plants after heat stress were also observed in the 5 mM CaCl_2_ treatment, compared with controls (Table 4 and Table 5). Goswami et al. [28] reported that a foliar spray of Ca^2+^ (10 mM) prior to heat stress (42 °C) at the grain-filling stage proved beneficial for the growth of wheat. Ca^2+^ ions protect cell membranes from heat injury, possibly by directly binding to cell membranes to reduce their fluidity under high temperatures. Therefore, these physiological parameters are suitable for evaluating the phenotypes of these plants under high-temperature stress and can help in the advanced interpretation of the photochemical process in sage plants. In particular, the SPAD, NVDI, Fv/Fm, and RI values of SO plants treated with 100 μM SA or 5 mM CaCl_2_ indicated increases in Chl content and photosynthetic capacity and reduced cell membranes to result in photoinhibitory effects, compared with controls. It is possible that Ca^2+^ and SA could be important modulators of the cellular signaling of transduction events following heat stress injury, and the development of short-term heat stress in leaves is more gradual or perhaps delayed by SA and CaCl_2_ treatments. However, these data still reflect the physiological attributes that contribute to our perception of plant ecophysiology and subsequent growth in outdoor planting sites.

The role of genetic variation is apparent from the observations of variation in the response of different species growing side-by-side in the same treatment and time duration. Plants adapt their photosynthesis to a certain degree in response to prevailing temperatures, and the sensitivity of photosynthesis to high temperatures varies between sage cultivars. When different chemical treatments across varieties were compared, SE plants exhibited higher NDVI values than SO plants under identical treatment at same time durations (Table 1, Table 4 and Table 6), implying that their genotypes exhibited different abilities and specificities in photosynthetic light-use efficiency. Thus, the NDVI of SE had better temperature tolerance than SO. The Fv/Fm reduction indicates that an important portion of the photosynthesis system (PS)II reaction center was damaged, as the Fv/Fm value in healthy, uninhibited leaves is typically 0.8.This value may be strongly depressed after exposure to heat stress, which precipitates the suppression of the electron transfer chain [29]. The highest Fv/Fm value (0.71) of SO plants with 100 μM SA treatment 3d after heat stress was close to 0.8 (Table 1), indicating that there was no long-term photoinhibition, and 30 °C was still suitable for the growth of these plants. However, lower Fv/Fm values were observed in untreated control plants, compared with chemically treated plants (Table 4 and Table 6). The photoinhibition of photosynthesis is characterized by a reduction in the quantum yield of photochemistry and reduced ChlF, which entails both the inhibition of PSII and increased thermal de-excitation of excited Chl [30]. Compared with controls, the pre-application of 100 μM SA + 5 mM CaCl_2_, followed by high temperature seemed to provide better adaptation to heat stress, and the induced heat stress tolerance may be directly linked to the coordinated response of Fv/Fm and the direct implication in the regulation of Chl content, which could help create better future agricultural methods in relation to current global warming predictions. In addition, lower RI% and better appearance led to greater heat alleviation in SO plants subjected to chemical treatments (except for 800 μM SA treatment), compared with untreated control plants (Table 2, Table 3, Table 5 and Table 7). Thermal damage to cell membranes was characterized by a marked increase in the RI%. High temperatures weaken cell membranes, which leads to the leakage of electrolytes from the cell.

Different photosynthetic parameters acted differently under heat stress and to SA and CaCl_2_ treatments; however, each index or chemical is not necessarily equally significant in protecting against heat stress. The impacts of changing these parameters on the sage species were affected by SA and CaCl_2_ applications, and different species displayed variations in their photosynthetic systems. The different expressions of each species were associated with the heat stress response, and these controlled environmental conditions may also be more practical for sage growers. Our results suggest that photosynthetic parameters were heat-stress specific and not expressed solely in response to an increasing excess of photon energy, and they are suitable for evaluating the morphology and physiology of specific genotypes subjected to specific chemical treatments. Therefore, combining RI, SPAD, NVDI, and Fv/Fm values resulting from chemical treatment after a high-temperature treatment in a growth chamber can be used to select against the most susceptible plants. In other words, these identified systems can be a more efficient use of land for evaluating new material in the field and used for rapid monitoring and early detection of heat injury in vegetative stages. This means that hundreds of individual chemical-treated plants grown under heat stress can be cost-effectively screened daily, providing ample opportunity to discover individuals that manifest better spectral reflectance and physiological indicators.

Optimal chemical applications improve leaf appearance and stabilize the quality of sage plants. It is essential to determine the precise treatments to maximize sage plant appearance and physiological reactions when applying a particular combination of chemicals. SA is involved in Ca^2+^-mediated signal transduction pathways in heat tolerance. Several studies established that Ca^2+^ cooperates with SA by enhancing various types of resistance in plants. Chen and Kuc [31] showed that Ca^2+^ was able to regulate both intracellular and extracellular transportation of SA. Chen et al. [32] reported that Ca^2+^ controlled the movement of SA in and out of plant cells. Guo et al. [33] demonstrated that SA treatment first led to Ca^2+^ release from internal stores of *Salvia miltiorrhiza*, and then a large amount of Ca^2+^ effluxed from apoplasts in cell culture. Lan et al. [34] illustrated that aluminum and SA increased cytosolic Ca^2+^ concentrations in soybean roots and SA-mediated cellular Ca^2+^ levels in soybean. Interestingly, the simultaneous addition of SA and Ca^2+^ reduced NaCl-induced oxidative stress in wheat more effectively by preventing MDA accumulation [22]. In our study, a strong synergistic interaction was observed between SA and CaCl_2_ regarding RI, SPAD, NVDI, and Fv/Fm levels, where maximum and significantly increased SPAD, NVDI, and Fv/Fm levels and reduced RI% of SO plants were detected in 100 μΜ SA and 5 mM CaCl_2_ combined treatments, compared with controls. Thus, the combined treatment has the ability to ameliorate heat stress and can be applied on a commercial scale to inform the development of rapid and precise management and integrated and quantitative measurements of bedded ornamental plants grown in plant factories or open fields to achieve maximum market benefit. Both SE and SO genotypes exhibita wide range of variability in appearance with desired chemical treatments and hence can be utilized directly or included in hybridization programs and prove useful in breeding programs for improving sage cultivars. For instance, subjecting SO plants to 100 μΜ SA and 5 mM CaCl_2_ can result in a better morphological appearance that would be useful for ranking cultivars according to photosynthetic capacity. The knowledge acquired from this study can be directly translated into specific management practices for re-vegetation, landscaping, xeriscaping, and urban greening.

## 4. Materials and Methods

### 4.1. Plant Materials and Culture Conditions

Sage plants SO species “Bergarrten” (shrubby with compact, woody stems and wrinkled gray-green leaves) and SE species “Scarlet Pineapple” (bushy sub-shrub with strongly pineapple-scented light green and ovate leaves) were obtained from local flower shops in New Taipei City, Taiwan, in July 2019. All plants were 16~19 cm tall and in the vegetative stage, transplanted into 9 cm plastic pots (200 mL, one plant per pot) containing commercial potting soil with a 4:1 (*v*/*v*) mixture of peat moss and perlite, and placed in a greenhouse at National Taiwan University (latitude 25.01° N), where the average temperature was 33.3 °C and the maximum temperature was 41.1 °C, with 14 h day lengths in July and August 2019. Plants were watered twice a week, and a compound fertilizer solution (20N-8.8P-16.6K water-soluble fertilizer at 1 g·L^−1^, Peter’s 20-20-20, Marysville, OH, USA) was applied weekly throughout the experiment. Plants were grown for two weeks, and those with uniform sizes were selected and randomly placed in an environment-controlled growth chamber under an irradiance of 200 μmol·m^−2^·s^−1^, a 14 h photoperiod, day/night temperatures of 30/25 °C, and relative humidity (RH) of 80% for 1 week. All plants were watered frequently to keep the growth substrate moist during the experiment. After that, 50 mL of different concentrations of SA (0, 100, 200, 400, and 800 μM) or CaCl_2_ (0, 5, 10, and 15 mM) alone were added to the soil of each pot twice a week in the late afternoon. Three hours after all plants manually received 100 mL of chemical treatments, the plants were exposed to 55 °C and 80% RH for 30 min, then moved to the growth chamber at 30 °C for 3 d, and the changes in phenotypic appearance and physiological measurements were analyzed at a different time interval. Chemical solution concentrations were selected based on data from our preliminary study screening experience. Plants given only water without the chemical solutions were used as controls. Six replicates (plants) per concentration per time interval per cultivar were used in each experiment. After investigating the differences in the two chemical treatments individually between cultivars, the optimal treatments of SA and CaCl_2_ were combined and fully mixed, and all test plants were subjected to the same experimental procedure as in the previous SA or CaCl_2_ individual experiments.

### 4.2. Determination of ChlF

Potted plants were moved into the shade under a cottage before sunrise at 05:30~06:00, acclimated to the dark for 20 min [35], and then ChlF parameters of their leaves were measured with a fluorometer (MINI-PAM, Walz, Effeltrich, Germany) at ambient temperature. The middle portions of the plants of both varieties were used for measurements. Values of the minimal ChlF (Fo) and maximal ChlF (Fm) of the dark-adapted samples were determined using the modulated irradiation of a weak LED beam (measuring light) and a saturating pulse. We then calculated the maximum photochemical quantum yield (Fv/Fm), where Fv, the yield of variable fluorescence, was calculated as (Fm-Fo). When measuring Fv/Fm, samples were first acclimated to dark conditions to ensure that all reaction centers were in an open state, and there was minimal nonphotochemical dissipation of excitation energy. ChlF data were collected at 0, 1, 2, and 3d of the experimental period after heat stress treatment, and measurements were recorded using WinControl-3 software (Heinz Walz, Effeltrich, Germany).

### 4.3. Determination of Spectral Reflectance

Spectral reflectance was measured from mature, healthy, fully expanded third leaves at wavelengths of 200~900 nm using an integrating sphere fitted to a scanning spectrophotometer (PolyPen RP 400, Photon Systems Instruments, Prague, Czech Republic) at 0, 1, 2 and 3 d after high-temperature treatment. The adjusted normalized difference vegetation index (NDVI) was calculated as (R750 − R705)/(R750 + R705 − 2 × R445) [35].

### 4.4. Measurement of Total Chl Content

Healthy, fully expanded mature leaves from the middle to upper portion of each plant were used to determine total chlorophyll content using a soil-plant analysis development (SPAD) analyzer (SPAD-502 Chlorophyll meter reader, Konica Minolta, Tokyo, Japan) at 0, 1, 2, and 3 d after heat stress [12].

### 4.5. Percent Elative Injury (RI%)

RI% was measured after high-temperature treatment as the conductivity of electrolyte leakage (EL) from the uppermost fully expanded leaf of each plant over a range of temperatures. Leaf discs 1 cm in diameter were treated by heating to 55 °C for 15 min. The heat treatment tubes were then cooled to 25 °C, and both the control and treatment tubes were filled with 15 mL of distilled water and incubated at 6 °C for 24 h to allow diffusion of electrolytes from the leaf discs. An initial conductance measurement (R1) was made using a conductivity meter (Model SC-170, SunTex, Taipei, Taiwan). Both the control and heat-treatment tubes were then autoclaved at 121 °C for 15 min to lyse all cells and release all their electrolytes, and final conductance measurements (R2) were taken. RI% induced from the initial 50 °C treatment was then calculated as follows: RI (%) = (R1/R2) × 100% [36].

### 4.6. Appearance of Landscape Performance

Throughout the experiment, after high-temperature treatment, the morphology of all chemical-applied and control plants in the vegetative stage was observed and rated as 1~5 points based on the level of heat-induced injury (Figure 1) [24]:

Level 1: all leaves had become brown, withered, and abscised;
Level 2: >60% of leaves had become brown, withered, and abscised;
Level 3: 30 ~ 60% of leaves had become brown, withered, and abscised;
Level 4: <30% of leaves had become brown and withered;
Level 5: normal growth.

### 4.7. Statistical Analysis

Data from appearance measurements, SPAD, NDVI, Fv/Fm, and RI were analyzed by analysis of variance (ANOVA) according to a completely randomized experimental design. Means were separated for each parameter using Fisher’s least significant difference (LSD) test at *p* *<* 0.05 by SAS ver. 9 (SAS Institute, Cary, NC, USA). The experiment was performed twice independently, with a randomized design for the growth environment, sampling day, and physiological analyses.

## 5. Conclusions

In this study, the following observations can be made:

(1) The influence of SA and CaCl_2_ treatments applied alone and in combination on sage plants was assessed by observing changes in the physiology and morphology of plants exposed to 30 °C for 3d. The exogenous application of SA and CaCl_2_ in optimal concentrations significantly and effectively enhanced Chl content and photosynthetic ability, and reduced cell membranes, in SO and SE plants, resulting in photoinhibitory effects, compared with controls at different time periods following heat stress. Thus, RI, SPAD, NVDI, and Fv/Fm can be used for screening sage plants for their growth and appearance following heat stress to produce sage plants that have high aesthetic and heat-tolerance qualities.
(2) A knowledge of heat-responsive parameters is critical for further understanding the physiological mechanisms of stress tolerance that can potentially be used to maximize the efficiency in the growth, development, and physiological potential of sage plants in controlledenvironments for economic benefit in industrial applications. The results can be used to improve the thermal intolerance of sage plants, and, in turn, used to develop management practices for its cultivation in gardens, reduce energy consumption, and enhance cultivation in hot and humid conditions during summer.
(3) SO plants treated with combined SA and CaCl_2_ showed more vigor than untreated controls, and new leaves of SO plants increased by facilitating SA- and CaCl_2_-mediated pathways for the accumulation of SA and Ca^2+^ in response to heat stress. Pretreatments with 100 μΜ SA and 5 mM CaCl_2_ can be made; moreover, when the weather forecast heatwave hits a week before, one can apply SA, CaCl_2,_ or a combination of both to protect sage plants, so that plants can survive the high-temperature period and reduce economic losses.

## Figures and Tables

**Table 1:** Effects of salicylic acid (SA) on the soil-plant analysis development (SPAD), normalized difference vegetation index (NDVI), and maximum photochemical quantum yield (Fv/Fm) values of *S. elegans* L. (SE) and *S. officinalis* Vahl (SO) in a greenhouse over a three-day period after heat stress treatment.

Species	SA		SPAD				NDVI				Fv/Fm		
		Day 0	Day 1	Day 2	Day 3	Day 0	Day 1	Day 2	Day 3	Day 0	Day 1	Day 2	Day 3
SE ‘Scarlet Pineapple’	Control	35.34 a	35.78 a	29.17 b	28.23 b	0.61 b	0.68 a	0.58 b	0.56 b	0.81 a	0.26 a	0.09 a	0.18 a
	100 µM	35.80 a	35.23 a	34.85 a	33.08 a	0.73 a	0.70 a	0.69 a	0.68 a	0.81 a	0.25 a	0.08 a	0.18 a
	200 µM	35.38 a	35.36 a	36.33 a	34.53 a	0.75 a	0.69 a	0.70 a	0.67 a	0.80 a	0.24 a	0.06 a	0.03 b
	400 µM	36.04 a	36.99 a	35.33 a	35.43 a	0.76 a	0.70 a	0.70 a	0.68 a	0.80 a	0.23 a	0.05 a	0.02 b
	800 µM	34.94 a	34.01 a	37.12 a	36.10 a	0.70 a	0.69 a	0.69 a	0.67 a	0.80 a	0.22 a	0.03 a	0.02 b
SO ‘Bergarrten’	Control	37.90 a	32.01 b	33.90 a	28.69 b	0.61 a	0.62 a	0.62 a	0.49 b	0.81 a	0.39 b	0.46 a	0.62 a
	100 µM	39.92 a	37.75 a	34.43 a	35.88 a	0.64 a	0.64 a	0.64 a	0.62 a	0.81 a	0.38 b	0.30 ab	0.71 a
	200 µM	38.33 a	38.23 a	35.13 a	34.36 a	0.65 a	0.65 a	0.63 a	0.63 a	0.81 a	0.49 a	0.38 ab	0.60 a
	400 µM	38.65 a	39.76 a	34.75 a	35.42 a	0.63 a	0.64 a	0.61 a	0.61 a	0.81 a	0.50 a	0.36 ab	0.52 ab
	800 µM	37.68 a	32.19 b	33.81 a	28.03 b	0.62 a	0.63 a	0.60 a	0.50 b	0.81 a	0.44 ab	0.23 b	0.38 b

Among various SA treatments of the same genotype within a column, means with the same lowercase letter do not significantly differ by the least significant difference (LSD) test at *p* < 0.05. Six replicates for each SA treatment of each cultivar were subjected to each concentration. Each treatment is assumed to be dependent on the other.

**Table 2:** Effects of salicylic acid (SA) on relative injury (RI) of *S. elegans* L. (SE) and *S. officinalis* Vahl (SO) in a greenhouse after high-temperature treatment.

Species	SA	RI (%)
SE ‘Scarlet Pineapple’	Control	40.2% a
	100 µM	30.6% c
	200 µM	36.5% bc
	400 µM	38.5% ab
	800 µM	42.8% a
SO ‘Bergarrten’	Control	48.7% a
	100 µM	26.8% c
	200 µM	28.1% c
	400 µM	36.5% b
	800 µM	53.9% a

Among various SA treatments of the same genotype within a column, means with the same lowercase letter do not significantly differ by the least significant difference (LSD) test at *p* < 0.05. Six replicates for each SA treatment of each cultivar were subjected to each concentration. Each treatment is assumed to be dependent on the other.

**Table 3:** Effects of salicylic acid and/or CaCl_2_ application on average grade of appearance of potted *S. elegans* L. (SE) and *S. officinalis* Vahl (SO) plants in a greenhouse three days after heat stress.

Species	Treatment	Grade of Appearance	Treatment	Grade of Appearance	Treatment	Grade of Appearance
SE ‘Scarlet Pineapple’	Control	4.9 a	Control	4.9 a	Control	4.7 a
	100 μM SA	4.9 a	5 mM CaCl_2_	4.8 a	5 mM CaCl_2_	4.8 a
	200 μM SA	5.0 a	10 mM CaCl_2_	5.0 a	100 μM SA	4.9 a
	400 μM SA	4.2 ab	15 mM CaCl_2_	4.9 a	100 μM SA + 5 mM CaCl_2_	5.0 a
	800 μM SA	3.9 b				
SO ‘Bergarrten’	Control	3.7 b	Control	3.0 c	Control	2.6 c
	100 μM SA	4.8 a	5 mM CaCl_2_	3.8 a	5 mM CaCl_2_	3.0 b
	200 μM SA	4.3 a	10 mM CaCl_2_	3.7 ab	100 μM SA	3.3 b
	400 μM SA	3.9 b	15 mM CaCl_2_	3.4 b	100 μM SA + 5 mM CaCl_2_	4.3 a
	800 μM SA	3.8 b				

Morphological quality was visually rated for plants in each pot on a scale of 1 to 5, with 5 indicating the best in all quality components and 1 indicating mostly brown, withered, and defoliated leaves. Among various treatments of the same genotype within a column, means with the same lowercase letter do not significantly differ by the least significant difference (LSD) test at *p* < 0.05. Six replicates for each chemical treatment of each cultivar.

**Table 4:** Effects of calcium chloride (CaCl_2_) on the soil-plant analysis development (SPAD), normalized difference vegetation index (NDVI), and maximum photochemical quantum yield (Fv/Fm) values of *S. elegans* L. (SE) and *S. officinalis* Vahl (SO) in a greenhouse over a three-day period after heat stress.

Species	CaCl_2_		SPAD				NDVI				Fv/Fm		
		Day 0	Day 1	Day 2	Day 3	Day 0	Day 1	Day 2	Day 3	Day 0	Day 1	Day 2	Day 3
SE ‘Scarlet Pineapple’	Control	35.00 b	34.25 a	30.99 b	29.00 b	0.69 a	0.63 a	0.57 a	0.57 a	0.80 a	0.05 b	0.04 a	0.04 a
	5 mM	39.50 a	38.85 a	38.50 a	38.54 a	0.68 a	0.63 a	0.63 a	0.62 a	0.80 a	0.19 a	0.07 a	0.06 a
	10 mM	39.00 a	37.68 a	39.00 a	38.55 a	0.65 a	0.64 a	0.62 a	0.63 a	0.80 a	0.18 a	0.08 a	0.07 a
	15 mM	35.01 b	35.96 a	37.93 ab	37.01 ab	0.64 a	0.62 a	0.62 a	0.63 a	0.81 a	0.17 a	0.05 a	0.05 a
SO ‘Bergarrten’	Control	36.05 a	39.19 a	38.96 a	34.25 a	0.60 a	0.61 a	0.61 a	0.48 b	0.81 a	0.38 a	0.09 c	0.27 b
	5 mM	35.86 a	35.00 b	36.19 b	36.01 a	0.60 a	0.60 a	0.61 a	0.58 a	0.80 a	0.41 a	0.43 a	0.50 a
	10 mM	35.16 a	34.96 b	33.33 b	35.96 a	0.60 a	0.60 a	0.60 a	0.56 ab	0.80 a	0.40 a	0.29 b	0.27 b
	15 mM	34.98 a	34.94 b	33.31 b	35.14 a	0.60 a	0.60 a	0.59 a	0.61 a	0.81 a	0.39 a	0.42 a	0.58 a

Among various CaCl_2_ treatments of the same genotype within a column, means with the same lowercase letter do not significantly differ by the least significant difference (LSD) test at *p* < 0.05. Six replicates for each CaCl_2_ treatment of each cultivar were subjected to each concentration. Each treatment is assumed to be dependent on the other.

**Table 5:** Effects of calcium chloride (CaCl_2_) on relative injury (RI) of *S. elegans* L. (SE) and *S. officinalis* Vahl (SO) in a greenhouse after high-temperature treatment.

Species	CaCl_2_	RI (%)
SE ‘Scarlet Pineapple’	Control	42.1 a
	5 mM	32.5 c
	10 mM	39.9 ab
	15 mM	38.9 b
SO ‘Bergarrten’	Control	56.7 a
	5 mM	46.1 b
	10 mM	39.0 c
	15 mM	34.6 c

Among various CaCl_2_ treatments of the same genotype within a column, means with the same lowercase letter do not significantly differ by the least significant difference (LSD) test at *p* < 0.05. Six replicates for each CaCl_2_ treatment of each cultivar were subjected to each concentration. Each treatment is assumed to be dependent on the other.

**Table 6:** Effects of SA and/or CaCl_2_ application on the soil-plant analysis development (SPAD), normalized difference vegetation index (NDVI), and maximum photochemical quantum yield (Fv/Fm) values of *S. elegans* L. (SE) and *S. officinalis* Vahl (SO) in a greenhouse over a three-day period after heat stress.

Species	Treatment		SPAD				NDVI				Fv/Fm		
		Day 0	Day 1	Day 2	Day 3	Day 0	Day 1	Day 2	Day 3	Day 0	Day 1	Day 2	Day 3
SE ‘Scarlet Pineapple’	Control	37.97 a	37.90 a	28.56 b	24.93 b	0.69 a	0.68 a	0.53 b	0.42 b	0.80 a	0.24 a	0.02 a	0.04 b
	5 mM CaCl_2_	37.85 a	37.81 a	38.46 a	29.00 ab	0.70 a	0.69 a	0.68 a	0.58 ab	0.80 a	0.23 a	0.03 a	0.10 ab
	100 µM SA	36.09 a	36.30 a	36.01 ab	35.48 a	0.69 a	0.68 a	0.69 a	0.68 a	0.80 a	0.22 a	0.04 a	0.13 ab
	5 mM CaCl_2_ + 100 µM SA	37.31 a	37.37 a	38.45 a	35.48 a	0.70 a	0.69 a	0.69 a	0.68 a	0.80 a	0.21 a	0.03 a	0.21 a
SO ‘Bergarrten’	Control	50.93 a	36.70 b	31.60 b	31.62 c	0.67 a	0.53 b	0.41 b	0.42 b	0.82 a	0.54 a	0.18 a	0.21 c
	5 mM CaCl_2_	50.00 a	50.00 a	31.59 b	31.62 c	0.67 a	0.68 a	0.42 b	0.41 b	0.82 a	0.54 a	0.18 a	0.36 b
	100 µM SA	48.02 a	51.11 a	52.00 a	48.93 b	0.67 a	0.68 a	0.67 a	0.67 a	0.82 a	0.53 a	0.18 a	0.50 ab
	5 mM CaCl_2_ + 100 µM SA	48.06 a	51.19 a	51.96 a	52.30 a	0.67 a	0.68 a	0.67 a	0.67 a	0.83 a	0.53 a	0.19 a	0.57 a

Among various SA and/or CaCl_2_ treatments of the same genotype within a column, means with the same lowercase letter do not significantly differ by the least significant difference (LSD) test at *p* < 0.05. Six replicates for each treatment of each cultivar were used. Each treatment is assumed to be dependent on the other.

**Table 7:** Effects of salicylic acid (100 μΜ) and/or CaCl_2_ (5 mM) applications on the relative injury (%) of *S. elegans* L. (SE) and *S. officinalis* Vahl (SO) in a greenhouse after high-temperature treatment.

Species	Treatment	RI (%)
SE ‘Scarlet Pineapple’	Control	43.9 a
	5 mM CaCl_2_	41.0 a
	100 µM SA	33.7 b
	5 mM CaCl_2_ + 100 µM SA	31.5 b
SO ‘Bergarrten’	Control	39.2 a
	5 mM CaCl_2_	29.1 b
	100 µM SA	28.9 b
	5 mM CaCl_2_ + 100 µM SA	25.7 b

Among various SA and/or CaCl_2_ treatments of the same genotype within a column, means with the same lowercase letter do not significantly differ by the least significant difference (LSD) test at *p* < 0.05. Six replicates for each treatment of each cultivar were used. Each treatment is assumed to be dependent on the other.
